# Precocious Sperm Exchange in the Simultaneously Hermaphroditic Nudibranch, *Berghia stephanieae*

**DOI:** 10.1093/iob/obac034

**Published:** 2022-08-01

**Authors:** Neville F Taraporevala, Maryna P Lesoway, Jessica A Goodheart, Deirdre C Lyons

**Affiliations:** Scripps Institution of Oceanography, University of California San Diego, 9500 Gilman Drive, La Jolla, CA 92093, USA; Scripps Institution of Oceanography, University of California San Diego, 9500 Gilman Drive, La Jolla, CA 92093, USA; Scripps Institution of Oceanography, University of California San Diego, 9500 Gilman Drive, La Jolla, CA 92093, USA; Scripps Institution of Oceanography, University of California San Diego, 9500 Gilman Drive, La Jolla, CA 92093, USA

## Abstract

Sexual systems vary greatly across molluscs. This diversity includes simultaneous hermaphroditism, with both sexes functional at the same time. Most nudibranch molluscs are thought to be simultaneous hermaphrodites, but detailed studies of reproductive development and timing remain rare as most species cannot be cultured in the lab. The aeolid nudibranch, *Berghia stephanieae*, is one such species that can be cultured through multiple generations on the benchtop. We studied *B. stephanieae* reproductive timing to establish when animals first exchange sperm and how long sperm can be stored. We isolated age- and size-matched individuals at sequential timepoints to learn how early individuals can exchange sperm. Individuals isolated at 10 weeks post initial feeding (wpf; ∼13 weeks postlaying [wpl]) can produce fertilized eggs. This is 6 weeks before animals first lay egg masses, indicating that sperm exchange occurs well before individuals are capable of laying eggs. Our results indicate that male gonads become functional for animals between 6 mm (∼6 wpf, ∼9 wpl) and 9 mm (∼12 wpf, ∼15 wpl) in length. That is much smaller (and sooner) than the size (and age) of individuals at first laying (12–19 mm; ∼16 wpf, ∼19 wpl), indicating that male and female functions do not develop simultaneously. We also tracked the number of fertilized eggs in each egg mass, which remained steady for the first 10–15 egg masses, followed by a decline to near-to-no fertilization. This dataset provides insights into the precise timing of the onset of functionality of the male and female reproductive systems in *B. stephanieae*. These data contribute to a broader understanding of reproductive development and the potential for understanding the evolution of diverse sexual systems in molluscs.

## Abrégé

Traduit par Maryna Lesoway et Hereroa Johnston

Les stratégies de reproduction sont énormément variables chez les mollusques. Cette diversité inclut les hermaphrodites simultanés pouvant être mâle et femelle à la fois. La plupart des mollusques nudibranches sont considérés être des hermaphrodites simultanés, mais les études détaillées du développement reproductif restent rares, car la plupart des nudibranches ne peuvent pas être cultivés en laboratoire. Le nudibranche aeolid, *Berghia stephanieae*, est l'une des rares espèces pouvant être facilement cultivées sur plusieurs générations sur une paillasse de laboratoire. On a étudié le développement temporel reproductif de *B. stephanieae* dans le but d‘établir à quel moment les individus font leurs premiers échanges de sperme et pour combien de temps ce dernier est gardé. Pour cela des individus ont été isolés en fonction de leur âge et de leurs tailles de manière séquentielle au cours de leurs développement afin de déterminer les premiers échanges de sperme.. Les individus isolés 10 semaines après avoir commencé de manger (∼13 semaines après avoir été pondu) sont capables de produire des œufs fertilisés. Cela se produisant 6 semaines avant que ces individus ne soient capables de déposer des masses d’œufs fécondés, indiquant que l‘échange de sperme a eu lieu bien avant que ces individus aient la capacité de pondre des œufs. Nos résultats indiquent que la gonade mâle devient fonctionnelle quand les individus mesurent entre 6 mm et 9 mm de longueur. Par contraste, ces individus ne pondent pas d’œufs avant de mesurer 12 à 19 mm de longueur, indiquant que les fonctions mâles et femelles ne commencent pas en même temps. De plus, on a compté le nombre d’œufs fécondés par masse d‘œufs, ce dernier restant inchangé pour les premières 10 à 15 masses d’œufs mais cela s'est suivi par un déclin rapide aboutissant à zéro œuf fécondé par masse d‘œuf. Les résultats présentés ici fournissent des informations précises à propos du début du fonctionnement des systèmes reproductifs mâle et femelle chez *B. stephanieae*. Ces données contribuent à une compréhension approfondie du développement reproductif avec le potentiel d’une meilleure compréhension de l’évolution des diverses systèmes de reproductions.

## Resumen

Traducción por Daniel Escobar-Camacho

Los sistemas sexuales varían ampliamente entre los moluscos. Esta diversidad incluye el hermafroditismo simultáneo, ambos sexos funcionales al mismo tiempo. Se cree que la mayoría de los moluscos nudibranquios son hermafroditas simultáneos, pero los estudios detallados del desarrollo reproductivo y su sincronización temporal son raros ya que la mayoría de las especies no se pueden mantener en el laboratorio. El nudibranquio eólido, *Berghia stephanieae*, es una especie que se puede mantener en cautiverio durante varias generaciones en condiciones de laboratorio. En este estudio, se analizó la sincronización del estado reproductivo de *B. stephanieae* para establecer el momento en el cual los animales intercambian esperma por primera vez y la duración de cuánto tiempo se puede almacenar el esperma. Para aprender cómo los individuos juveniles intercambian esperma, aislamos individuos de la misma edad y tamaño, en diferentes puntos de tiempo en una secuencia temporal. Se pudo observar que los individuos aislados a las 10 semanas, después de la primera alimentación (wpf; ∼13 semanas después de la puesta, wpl), pueden producir huevos fertilizados. Esto es 6 semanas antes de que los animales desoven masas de óvulos por primera vez, lo cual sugiere que el intercambio de esperma ocurre mucho antes de que los individuos sean capaces de desovar óvulos. Nuestros resultados indican que las gónadas masculinas se vuelven funcionales para animales de entre 6 mm (∼6 wpf, ∼9 wpl) y 9 mm (∼12 wpf, ∼15 wpl) de longitud. Este tamaño es más pequeño (y más temprano) que el tamaño (y la edad) de los individuos en su primera puesta de huevos (12–19 mm; ∼16 wpf, ∼19 wpl), lo que indica que la funcionalidad de machos y hembras no se desarrollan simultáneamente. También analizamos la cantidad de huevos fertilizados en cada masa de huevos, que se mantuvo constante durante las primeras 10 a 15 masas de huevos, seguido de una disminución de fertilización hasta casi ser nula. Estos datos proporcionan información sobre el momento preciso del inicio de la funcionalidad de los sistemas reproductivos masculino y femenino en *B. stephanieae*, y contribuyen a una comprensión más amplia del desarrollo reproductivo y la evolución de los diversos sistemas sexuales en los moluscos.

## Introduction

Sexual systems—the patterns of sex allocation within a species—are diverse across animal lineages. While most animals have separate sexes (gonochorism, dioecy), hermaphroditism (monoecy) occurs in 5% of all described species, increasing to 30% when insects are excluded (Jarne and Auld 2006). Most major metazoan lineages have evolved hermaphroditism, which includes two major categories: simultaneous and sequential hermaphroditism, with female and male function occurring either at the same time or at different times, respectively. Current hypotheses suggest that reproductive plasticity plays an important role in the evolution of sexual systems (Leonard 2013, 2018).

Although definitions of hermaphroditism imply sharp distinctions between sequential and simultaneous hermaphroditism, the underlying biology is not so clear-cut (Heller 1993; Collin 2013; Leonard 2018). Apportioning of reproductive output between male and female functions in simultaneous hermaphrodites often changes in response to environmental inputs, including social and physical environment (Tomiyama 1996; Yusa 1996; Lorenzi et al. 2006; Baeza 2007; Janicke and Schärer 2009; Ramm et al. 2019). However, relatively little empirical work has addressed the biology of simultaneous hermaphrodites in comparison to studies of separate sexes, particularly in groups where transitions among different sexual systems are more common. In order to understand how transitions between sexual systems might occur, more detailed anatomical and reproductive data are needed (Nakadera and Koene 2013). In particular, longitudinal reproductive data in simultaneous hermaphrodites, including how and when reproductive resources are allocated, will be important to understanding transitions between sexual systems.

Molluscs are an excellent group in which to study reproductive diversification and the mechanisms underlying transitions between sexual systems. Molluscan sexual systems are highly labile and include multiple origins of simultaneous and sequential hermaphroditism, with the greatest diversity of sexual systems in molluscs found in gastropods and bivalves (Collin 2013, 2018; Lesoway and Henry 2019). Within gastropods, separate sexes are thought to be ancestral, and both simultaneous and sequential hermaphroditism have evolved repeatedly (Collin 2013). For example, all members of the family Calyptraeidae are sequential hermaphrodites (Collin 2013, 2018; Lesoway and Henry 2019), and the subclass Heterobranchia, which includes both land snails (pulmonates) and sea slugs (e.g., nudibranchs), is considered (primarily) simultaneously hermaphroditic (Heller 1993; Jarne and Auld 2006).

**Fig. 1:**
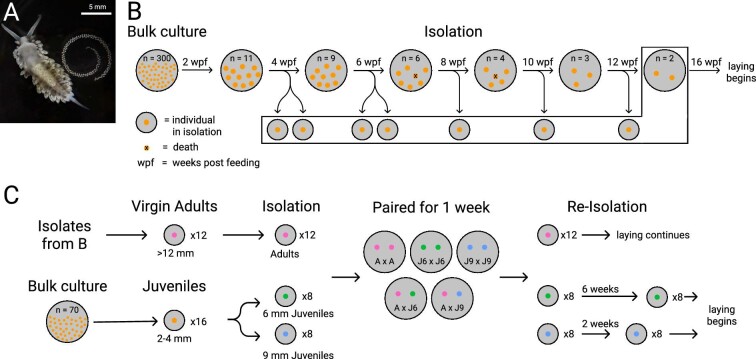
Experimental design for reproductive isolation experiments. (**A**) Adult *B. stephanieae* in ventral view, with egg mass. Scale = 5 mm. (**B**) Approximate age and size-matched juveniles from the initial bulk culture were separated into 6 groups of 11 animals (*n* = 66 total individuals), which were reared for a total of 23 weeks. Juveniles (*n* = 40) were then sequentially isolated over a period of 8 weeks. Animals began laying egg masses at around 16 wpf. On average, 2–3 individuals died from each group over the isolation period. (**C**) New individuals and virgin adults from the first experiment (**B**) were grown in isolation, paired for 1 week, then re-isolated. Previously isolated adults continued to lay egg masses, and juvenile animals began laying egg masses after 2–6 weeks of continued growth. In both experiments, egg masses produced by isolated animals were collected and fertilized and unfertilized eggs were counted. Colored circles indicate single individuals and x indicates dead individuals.

Within Heterobranchia, nudibranchs have proven to be useful for studies of development (Bonar and Hadfield 1974; Carroll and Kempf 1990; Kempf et al. 1997), animal behavior and neurobiology (Willows 1971; Kriegstein et al. 1974; Chase 2002; Katz and Quinlan 2019), nematocyst sequestration (Goodheart and Bely 2017; Goodheart et al. 2018, 2022), and reproduction (Rutowski 1983; Rivest 1984; Hall and Todd 1986; Sekizawa et al. 2019). Nudibranchs and other heterobranchs are typically described as simultaneous hermaphrodites with concurrently functioning male and female sex organs and reciprocal sperm exchange between conspecifics during mating (Hall and Todd 1986; Karlsson and Haase 2002; Sekizawa et al. 2019). However, descriptions of reproduction in most hermaphroditic species are often based on limited sampling of a few adult individuals (Collin 2013), or are limited to descriptions of reproductive behaviors. Several sources describe nudibranchs as having a more complex sexual system than simply simultaneous hermaphroditism (Tyrell Smith and Carefoot 1967; Harris 1975; Todd et al. 1997; Wägele and Willan 2000). Indeed, the diversity of sexual systems appears to be something of an open secret in the nudibranch literature (Hadfield and Switzer-Dunlap 1984; Heller 1993; Wägele and Willan 2000), but supporting data remains limited to a single nudibranch species, *Phestilla sibogae* (Todd et al. 1997). Evidence from heterobranch sea slugs is similarly sparse, but includes documented cases in the saccoglossan *Alderia modesta* (Angeloni 2003) and the cephalaspid *Mariaglaja tsurugensis* (=*Chelidonura sandrana*) (Sprenger et al. 2009). Studies that address the timing of gonadal development in nudibranchs (and other heterobranchs) remain rare, leaving open many questions about how and when reproductive resources are allocated in members of this group. However, our understanding of nudibranch biology is limited by the challenges of culturing nudibranchs through the complete life cycle in the lab. The aeolid nudibranch, *Berghia stephanieae* (Fig. 1A), can be kept easily in the lab, and presents a convenient research organism for studying nudibranch reproduction and development (Carroll and Kempf 1990; Kristof and Klussmann-Kolb 2010; Dionísio et al. 2013).

The availability of long-term cultures of *B. stephanieae* allows us to ask questions about when sperm is produced and exchanged, how sperm is allocated, and allows us to analyze changes in reproductive output over time. Here, we ask: (1) how early are individuals able to exchange sperm and can individuals exchange sperm at the same time that they produce fertilized eggs; (2) how long are isolated individuals able to retain sperm; and (3) how does sperm quality change over time? To determine the earliest stage at which these animals exchange sperm, we separated individuals at sequential timepoints. To confirm our results, we also performed reciprocal mating experiments on isolated individuals of different sizes, in addition to confirming our observations with histological sectioning of reproductive tissues across multiple reproductive timepoints. Together, our results show that individuals can produce and exchange sperm prior to producing eggs, documenting an additional species of nudibranch with individuals that function first as males, then as simultaneous hermaphrodites. In addition, we report longer sperm storage times than previously reported for other nudibranchs, and document changes in fertilization over time.

## Methods

### Adult cultures and juvenile collection

Adults of *Berghia stephanieae* (Valdés 2005) (Fig. 1A) were originally acquired from Reeftown (reeftown.com), and then maintained in continuous culture in our laboratory. Briefly, animals were kept in large finger bowls in artificial seawater at a salinity of 1.024 sg (Instant Ocean, Spectrum Brands, Blacksburg, VA) at room temperature (∼20°C) and allowed to mate freely. Groups and individuals were fed enough *Exaiptasia diaphana* (Carolina Biological Supply, Burlington, NC) to develop properly (see Supplementary File 1), and in order to prevent cannibalism.

### Age-based isolation

In our first experiment, juveniles of a known age were used for controlled matings. First, egg masses were collected within a five-day window and allowed to hatch and metamorphose (∼2–3 weeks postlaying [wpl]) into juveniles in a large fingerbowl. A group of ∼300 of these recently metamorphosed juveniles (∼3 wpl) were then fed together prior to experimental isolation. In these cultures, development is asynchronous, as larger animals consuming most of the food can limit growth of smaller individuals (Monteiro et al. 2020). To minimize such asynchronous development, after 2 weeks of feeding (2 weeks postfeeding [wpf]), the 66 largest individuals (∼1–2 mm long) were removed from the bulk culture in the finger bowls and raised in 6-well plates (Corning, #3736), grouped by size into 6 subgroups of 11 individuals each (Fig. 1B). At 4 wpf (∼7 wpl), 2 individuals from each subgroup (12 total) were isolated to their own separate wells to prevent access to additional mating events. Subsequently, 12, 6, 6, and 4 individuals were likewise each separated into individual wells at 6, 8, 10, and 12 wpf, respectively. The remaining 4 individuals were kept in 2 mating pairs that continued to have mating access for the duration of the experiment (Fig. 1B).

All individuals, regardless of mating status, start producing egg masses at 16 wpf (Fig. 1B). Isolated and grouped animals were maintained for an additional 5 weeks (21 wpf) to give them sufficient time to lay several egg masses (∼16 wpf; ∼19 wpl). When individuals began to lay eggs, egg masses from each animal were collected daily. Egg masses from animals isolated at 12 wpf were continuously collected until egg mass production ceased, after 16 weeks in total. Over the course of the experiment, a third of the animals cultured (22 individuals) died, leaving 44 animals for isolation experiments (Fig. 1B). Individuals that were isolated but died (*n* = 7) before laying any egg masses were not included in our analyses.

**Fig. 2:**
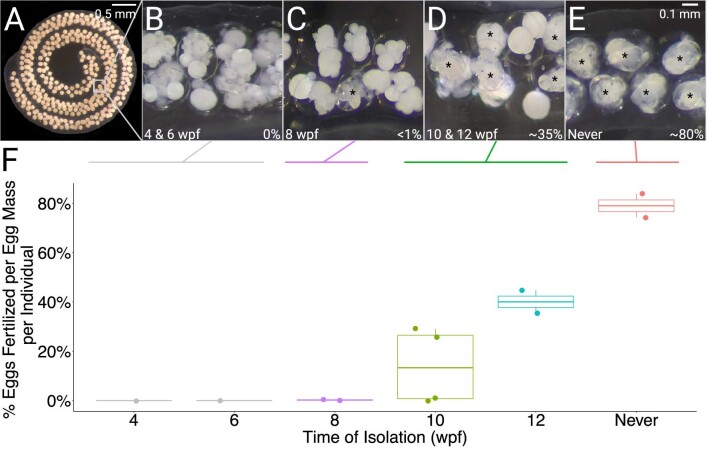
Later isolation increases the proportion of fertilized eggs per egg mass. (**A**) Coiled egg mass of *B. stephanieae* containing multiple encapsulated embryos. (**B**) Unfertilized eggs from an egg mass laid by an animal isolated at 6 wpf. (**C**) One fertilized egg among unfertilized eggs from an egg mass laid by an animal isolated at 8 wpf. (**D**) Some fertilized eggs in an egg mass laid by an animal isolated at 12 wpf. (**E**) Most eggs are fertilized in an egg mass laid by an animal that was never isolated. (**F**) Average proportion of fertilized eggs per egg mass laid by age-isolated animals, grouped by time of isolation. Each point represents all egg masses produced by an individual. Asterisks (**B–E**) indicate fertilized embryos. Scale: A = 0.5 mm, B–E = 0.1 mm.

**Fig. 3:**
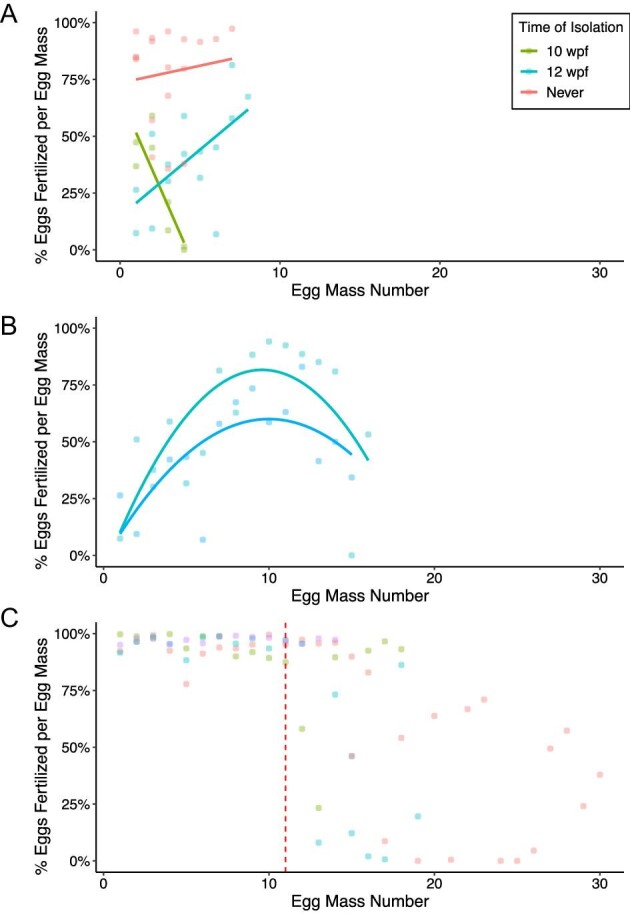
Proportion of fertilized eggs changes over time in both time- and size-isolated individuals. (**A**) Proportion of fertilized eggs laid by age-isolated animals over the 5-week experimental period. Egg masses are grouped by the time individuals were isolated (**B**) Proportion of fertilized eggs laid by two individuals isolated at 12 wpf over a 12-week period (continued from A). (**C**) Proportion of fertilized eggs in egg masses produced by large juveniles mated with large juveniles (9 mm, LJxLJ) in the size selection experiment. There is a sharp decrease in the proportion of fertilized eggs at the 11th egg mass (red line).

Egg masses were removed from plates with forceps and separated into 12-well plates (ThermoScientific, #150200) in ∼3 mL of 0.22 μm filtered artificial seawater for 1–2 weeks and allowed to develop. Previous experiments in the lab confirmed these as optimal conditions for development, with typically low mortality in developing embryos (>80% metamorphosis). Embryos were then examined and categorized into unfertilized eggs or fertilized embryos, based on their morphology. Embryos that showed stereotypical cleavage and developed into typical veligers (Fig. 2E) were categorized as fertilized. All others including non-cleaving- and chaotic-cleaving-eggs (Fig. 2B) were categorized as unfertilized. In egg masses containing fertilized eggs, all eggs, unfertilized and fertilized, were counted (Fig. 3). Egg masses without fertilized eggs were scored as being unfertilized but the number of eggs was not counted.

### Size-based isolation

**Fig. 4:**
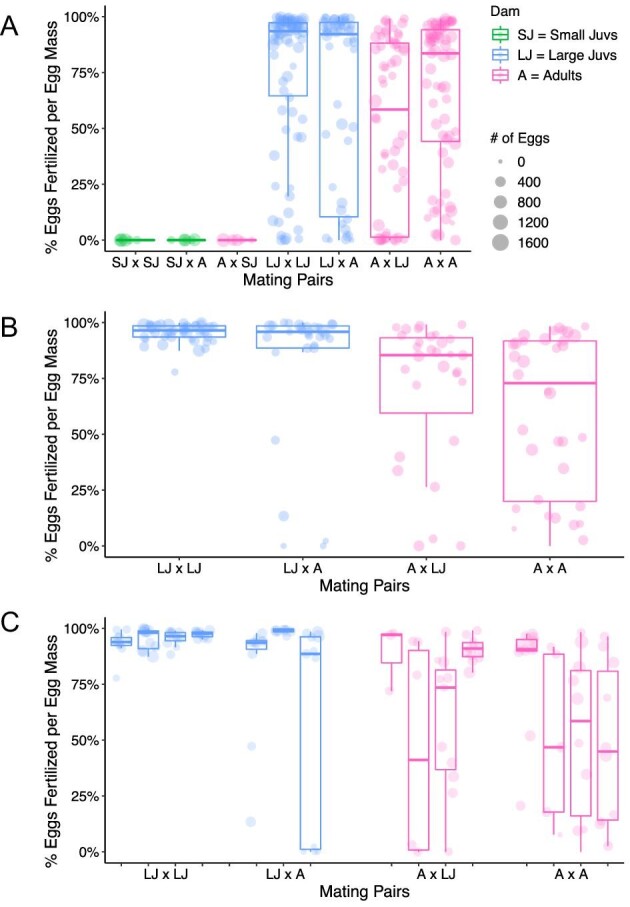
Proportion of fertilized eggs produced by size-isolated individuals. (**A**) Proportion of fertilized eggs in all egg masses laid by all animals mated by size. The first individual listed in each cross is the animal that laid the egg mass (dam), and second is the mate. (**B**) The proportion of fertilized eggs in the first 10 egg masses laid by adults and large juveniles, categorized by mate. Each plot represents the average of all animals in each treatment. (**C**) The proportion of fertilized eggs in the first 10 egg masses laid by individual adults and large juveniles, categorized by mate. Each box plot represents the egg masses laid by each individual.

Development in *B. stephanieae* is highly asynchronous and varies depending on environmental conditions (i.e., food availability, temperature, and inter-individual feeding competition), making estimations of age in bulk culture challenging. As demonstrated above, tracking a precise age is time-consuming and labor-intensive. We therefore performed a second experiment isolating animals by size (Fig. 1C), which can be assessed easily. Based on the results of our first experiment, we estimated size classes of animals likely to be able to produce and exchange sperm. We determined three size categories of animals, which we termed small juveniles (5–6 mm, equivalent to animals from age-based experiment ∼6 wpf/∼9 wpl, Fig. 4A), large juveniles (over 9 mm, ∼12 wpf/∼15 wpl, Fig. 4B), and adults (>12 mm, >16 wpf/>19 wpl, Fig. 4C). We expected small juveniles not to be able to exchange sperm, and large juveniles and adults to exchange sperm. Unmated adults (*n* = 12) were taken from our previous experiment. These adult animals, isolated at 4 and 6 wpf, were presumed to have never received sperm since they laid only unfertilized egg masses and were over 15 mm in length. The juveniles (2–4 mm in length, *n* = 16) were selected from a new bulk culture and put into isolation until they reached the appropriate size for our experiment (5–6 mm and >9 mm; Fig. 1C).

Animals were measured from photographs taken while fully extended during active crawling, from the anterior-most part of the head to the furthest posterior cerata (Fig. S1). Photographs of each animal were taken using a dissecting scope (Zeiss Stemi 305) and a Google Pixel 3A smartphone. Five separate images were taken of each animal, and the length of the animal was measured in each of these images using Fiji (Schindelin et al. 2012).

In total, 14 pairs were mated, including 2 pairs each of virgin adults, small juveniles, and large juveniles, and 4 pairs each of virgin adults with either small or large juveniles (Fig. 1C). Paired animals were allowed to mate for 7 days and were not fed to minimize growth. When two animals of the same category were paired, a small clump of cerata (finger-like projections of the dorsum) was removed from one animal to differentiate the two individuals. The animals were then re-isolated and fed one-twelfth of a medium-sized *E. diaphana* every other day for the remainder of the experiment (2–4 months). Adults (>7 months postlaying) began laying egg masses immediately, while large and small juveniles (>2 months postlaying) started laying egg masses ∼2 and ∼6 weeks after re-isolation, respectively (Fig. 1C). Egg masses were collected following the same procedure as described above. Additionally, one juvenile and two adults were kept in isolation for the duration of the experiment as controls.

### Statistical analyses

All statistical analyses were performed using R (v.3.6.2) (R Core Team 2014). The relationship between the fertilization rate of egg masses (dependent variable) and the order of egg masses laid (independent variable) was analyzed using a generalized linear model (glm function in stats; Fig. 3A) with time-based isolation treatment (10 wpf, 12 wpf, and never isolated individuals), the interaction of fertilization rate * treatment, and individual as additional fixed effects. For the size-based experiments, differences in fertilization rates among mating categories (Fig. 4A and B) were analyzed using the anova function in stats (Fig. 4A and B) with individual as an additional fixed effect, followed by Tukey HSD tests to assess which specific mating category means were significantly different. Individual variation within mating categories was then analyzed with the kruskal.test function in stats (Kruskal–Wallis Rank Sum Test; Fig. 4B and C) due to heteroscedasticity in the data (*P* < 0.0001 for adults and juveniles based on Levene's Test, leveneTest function in stats). Graphs were prepared using ggplot2 (v. 3.3.6) (Wickham 2009). Figures were prepared using Figma (figma.com) and Adobe Illustrator (v26.3.1). All relevant scripts and raw data files are available on GitHub (https://github.com/lyons-lab/berghia_reproduction).

### Histology and imaging

Animals matching the previously determined size categories (6 mm, 9 mm, and >12 mm) were measured and selected from bulk cultures, as described above. Animals were relaxed in a 1 part 7.5% MgCl_2_ to 2 parts Artificial Sea Water (ASW) solution until movement ceased, and then fixed in 4% paraformaldehyde in filtered sea water solution overnight at 4°C. A postfixation stain was then performed using Ponceau S (0.1% Ponceau S and 1.0% glacial acetic acid) for 2 h, followed by a diH_2_O rinse. The tissues were then dehydrated: (1) 50% ethanol (EtOH) for 15 min, (2) 60% EtOH for 15 min, and (3) 70% EtOH for 15 min. Tissues were then stored in 70% EtOH (at 4°C) or immediately prepared for embedding. In preparation for embedding, we subjected tissues to a further dehydration series: (1) 80% EtOH for 15 min, (2) 95% EtOH for 15 min, and (3) 100% EtOH for 15 min (x2). Samples were embedded with Spurr's Low Viscosity Embedding Media Kit (Electron Microscopy Sciences #14,300), following the “Firm” formulation provided by the manufacturer, and cured at 60°C overnight. Sections 3 µm thick were cut using glass knives on a microtome, and sections were mounted and counterstained with Azure A. Sections were imaged using a Zeiss AxioM2 fluorescence microscope with an attached digital camera and ZEN software (v2.3). Image stacks were combined in Helicon Focus (v7.7.5), adjusted for brightness and contrast using Fiji (v1.53f51, Schindelin et al. 2012). Image plates were prepared using the FigureJ plugin (Mutterer and Zinck 2013), https://imagej.net/plugins/figurej), and Adobe Illustrator (v26.0.3).

## Results

### Age-based isolation: later isolation increases fertilization rates

Successive isolation of carefully age-and size-matched *B. stephanieae* juveniles indicates the approximate age that individuals are able to exchange sperm (Fig. 2). The earliest isolated individuals (4 and 6 wpf) laid only unfertilized eggs (*n* = 42 egg masses, Fig. 2B). Of the 15 egg masses (>1000 eggs total) laid by animals isolated at 8 wpf, only a single egg was fertilized (Fig. 2C). Juveniles isolated at 10 and 12 wpf consistently produced fertilized eggs (Fig. 2D), laying 10 and 15 egg masses, respectively, with all but 2 containing fertilized eggs. Animals that were never isolated laid 17 egg masses, all of which contained fertilized eggs (Fig. 2E).

Fertilization rates varied among treatments, and animals with continuous access to sperm had significantly higher fertilization rates compared to individuals isolated at 10 and 12 wpf (*P* < 0.001 for each). For animals that were never isolated, average fertilization was high (78.0 ± 21.1%, Fig. 2E and F), while animals isolated at 10 and 12 wpf laid egg masses with low average fertilization rates (24.3 ± 23.2% and 39.8 ± 22.0%, Fig. 2D and F). The long duration of these experiments also allowed us to observe changes in fertilization rates over time (r^2^ = 0.678; *P* < 0.001, Fig. 3A). For the animals that were never isolated, the proportion of eggs fertilized in each egg mass did not significantly change over time (*P* = 0.906, Fig. 3A). Two animals isolated at 10 wpf laid multiple fertilized egg masses. In these animals, fertilization declined significantly (*P* = 0.012, Fig. 3A). For the two animals isolated at 12 wpf, fertilization rates measured during the initial 5-week experimental period increased (*P* = 0.011, Fig. 3A). We therefore continued to measure fertilization rates in these animals for a further 7 weeks. Over the course of 13 weeks, fertilization rates of egg masses laid by animals isolated at 12 wpf best fit a quadratic equation (r^2^ = 0.525; *P* < 0.001, Fig. 3B), with the highest fertilization rate around the 10th egg mass laid (Fig. 3B).

### Size-based isolation: large juveniles are able to exchange sperm with adults and each other

Asynchrony in juvenile development in *B. stephanieae* makes estimating age difficult, so in order to make our results more broadly applicable to our batch cultures, we turned to size as a proxy for reproductive stage. Guided by our previous experimental results, we defined three size classes: small juveniles (6 mm long), large juveniles (9 mm long), and adults (>12 mm long). Small juvenile pairings (<6 mm at the time of mating) produced almost no fertilized egg masses (only a single egg was fertilized out of 28 egg masses, Fig. 4A; SJxSJ). Small juveniles paired with adults produced almost no fertilized eggs (Fig. 4A; SJxA), with the exception of a single fertilized egg produced by one animal in the second of 3 otherwise unfertilized egg masses. Similarly, adults paired with small juveniles did not produce any fertilized eggs (Fig. 4A; AxSJ). Pairings of large juveniles (9 mm; LJxLJ) and adult hermaphrodites (>12 mm; AxA) produced primarily fertilized egg masses (Fig. 4A). Overall, both adult hermaphrodites and large juveniles were able to exchange sperm, while small (immature) juveniles were not.

The proportion of fertilized eggs per egg mass produced when animals were isolated based on size significantly correlated with the size of the animal laying the egg mass. As the proportion of fertilized eggs dropped significantly starting around the 12–15th egg mass laid (red line, Fig. 3C), presumably due to animals “running out of sperm,” we considered only the first eleven egg masses produced by each animal to test for differences in fertilization between adult hermaphrodites and large juveniles. The proportion of fertilized eggs laid by adult hermaphrodites mated with other adult hermaphrodites was the lowest (not including small juveniles) and most variable (59.1% ± 35.5%, Fig. 4B), followed by adult hermaphrodites mated with large juveniles (69.6% ± 34.1%), and large juveniles mated with adult hermaphrodites (84.6% ± 30.0%). Large juveniles mated with other large juveniles had the highest proportion of fertilized eggs and were the least variable (95.5% ± 4.3%, Fig. 4B). Large juvenile dams produced egg masses with higher proportions of fertilized eggs than adult hermaphrodite dams regardless of whether egg masses were sired by adults (LJxA versus AxA; *P* = 0.013, Fig. 4B) or large juveniles (LJxLJ versus AxLJ; *P* < 0.001, Fig. 4B), or when large juveniles mated with large juveniles and adults mated with adults (LJxLJ versus AxA; *P* << 0.001, Fig. 4B). However, large juveniles mated with adults did not produce significantly different proportions of fertilized eggs than adults mated with large juveniles (*P* = 0.733, Fig. 4B). Regardless of whether the sire was an adult or a large juvenile, the proportion of fertilized eggs was similar for egg masses laid by adult hermaphrodites (*P* = 0.702, Fig. 4B) and large juveniles (*P* = 0.128, Fig. 4B). As noted, the % of eggs that are fertilized in each egg mass remains steady for

the first ∼11 egg masses on average, followed by a decline to near-to-no fertilization (Fig. 3C). While the proportion of fertilized eggs varied based on treatment, in all cases, fertilization eventually declined to zero.

Within groups, individuals were fairly consistent in egg mass fertilization (Fig. 4C). Among the first 11 egg masses laid, adult hermaphrodites (*n* = 8) had consistently high variation. A Kruskal–Wallis test suggested differences among individuals (*P* = 0.010), but a Dunn test showed that no individual was significantly different from another (Fig. 4C). Large juveniles (*n* = 7) generally produced egg masses with consistently high fertilization rates, and a Kruskal–Wallis test suggested differences among the individual juveniles. A Dunn test showed only one individual (T5) was significantly different from other samples (Fig. 4C). This indicates that there is little variation among individuals within a group, even though adult hermaphrodites generally show reduced fertilization when compared to large juveniles.

### Histology: gonadal development and sperm localization

**Fig. 5:**
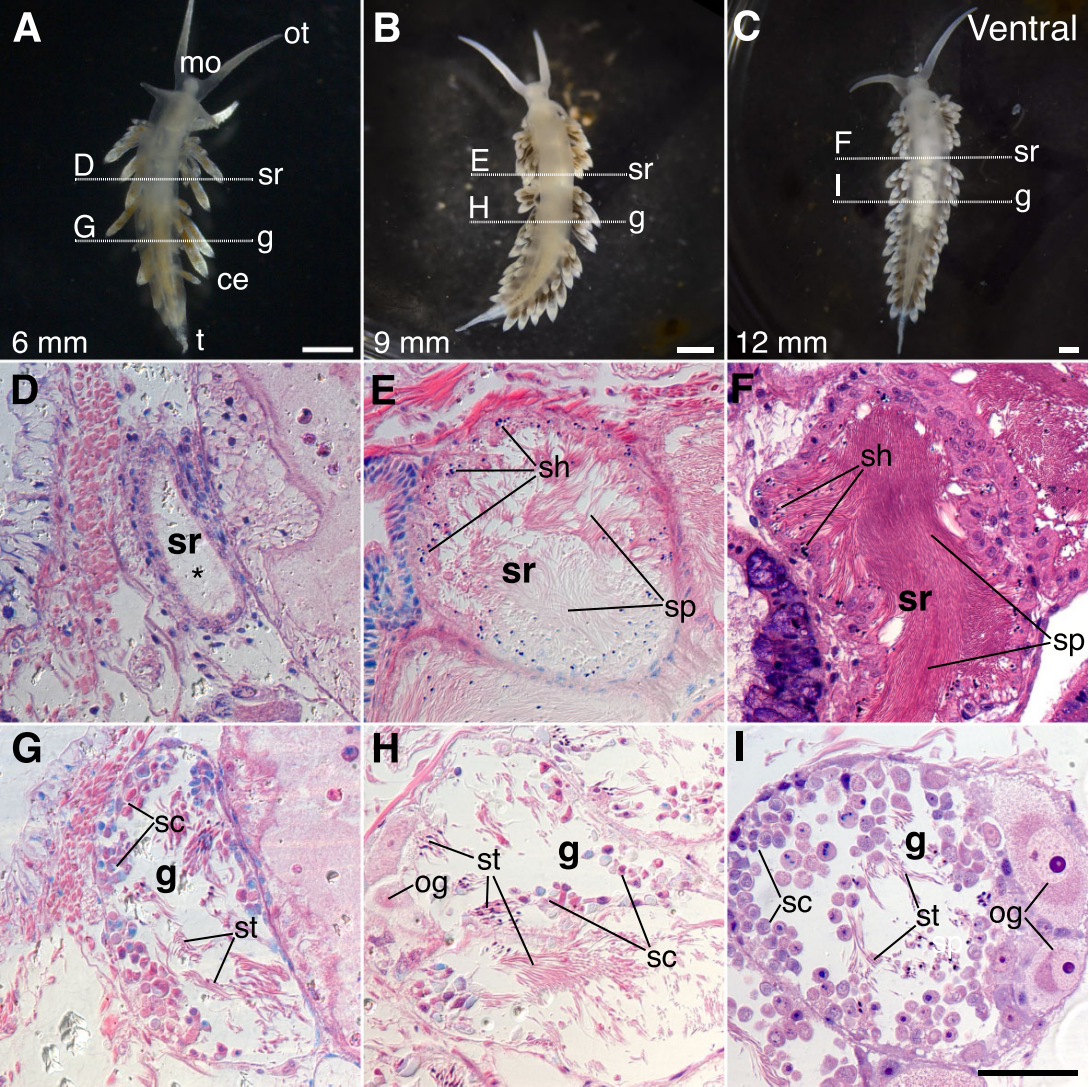
Histology of reproductive development in *B. stephanieae*. (**A)–(C**) Ventral views of 6 mm **(A)**, 9 mm (**B**), and 12 mm **(C)** juveniles of *B. stephanieae*. Horizontal lines indicate the approximate level of histological sections. (**D)–(F**) Sections cut through the seminal receptacles show increasing amounts of stored allosperm. Sperm heads are directed toward the wall, in contrast to autosperm, which are randomly oriented within the ampulla (data not shown). No sperm was seen in the reproductive tract of the 6 mm animal (**D,** asterisk) outside of the gonad (**G**). (**G)–(I)** Histological sections cut through the developing gonads. Both spermatocytes and maturing oocytes are produced in the ovotestis. Sperm maturation **(G**) precedes oocyte maturation **(I)**. ce, cerus; g, gonad; mo, mouth; t, tail; ot, oral tentacle; sh, sperm heads; sp, sperm; st, spermatid; sc, spermatocyte; sr, seminal receptacle; oc, oocyte. Scale: A–C = 1 mm, D–I = 50 μm.

Mating experiments using either age- or size-selected animals (above) indicate that sperm exchange occurs prior to fertilization and egg mass laying. To confirm the presence and location of sperm in the reproductive tract and the timing of sperm and egg development, we cut histological sections of small juveniles, large juveniles, and adults as determined by size (see above). Histological sections showed clear differences in gonadal maturation across the small juvenile (6 mm; Fig. 5A), large juvenile (9 mm; Fig. 5B), and adult (>12 mm; Fig. 5C) stages (Fig. 5D–I). At all stages, the gonad (or ovotestis), where sperm (i.e., autosperm) and eggs are produced, contains mature sperm and sperm progenitor cells (Fig. 5G–I). The ovotestis of adult hermaphrodites also contained mature oocytes (Fig. 5I), and signs of oocyte maturation were visible in large juveniles (Fig. 5H). Mature oocytes were not observed in the reproductive tract of the individuals sectioned. Reproductive morphology of nudibranchs is complex, with structures for storage of autosperm (ampulla, data not shown) and allosperm, sperm received from mates (seminal receptacles) (Fig. 5D–F). Identification of these structures often relies on location in the animal and orientation of sperm. Autosperm are typically oriented at random within the ampulla, while allosperm are oriented with the sperm heads directed into the epithelium of the seminal receptacle (Wägele and Willan 2000). No sperm was observed outside the ovotestis in the 6 mm juveniles (Fig. 5D), while the seminal receptacles and ampullae of both large juveniles and adults contained sperm (Fig. 5E and F). Together, the presence of sperm (both autosperm and allosperm) outside the gonad in large juveniles and the delayed maturation of oogonia confirm that sperm production and exchange precede egg maturation and fertilization in *B. stephanieae*.

## Discussion

**Fig. 6:**
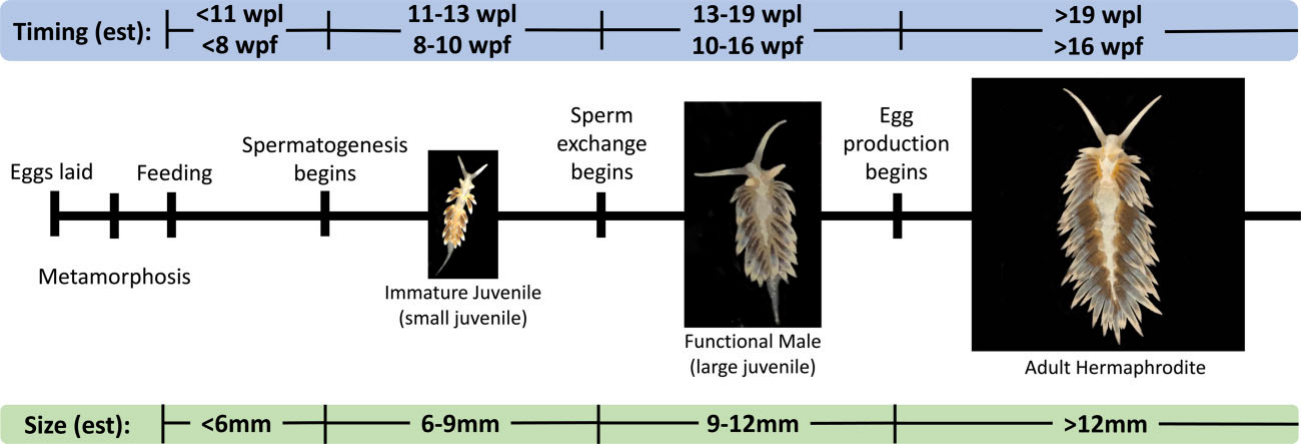
Summary of *B. stephanieae* reproductive development, with estimated timing at RT (∼20°C) and approximate size ranges (mm). Small juveniles are functionally immature, (∼8–10 wpf; ∼11–13 wpf; ∼6–9 mm in size) are able to produce sperm but are unable to exchange sperm. Large juveniles (∼10–16 wpf; ∼13–19 wpl; ∼9–12 mm in size) are functionally male and are able to both produce and exchange sperm. Hermaphroditic adults (>16 wpf; >19 wpl; >12 mm in size) have both male and female function and are able to produce and exchange sperm and lay fertilized egg masses. est, estimated; mm, millimeters.

Our study shows that *B. stephanieae* are not simply simultaneous hermaphrodites and highlights the complicated nature of sexual systems and their development. Male and female functions do not develop simultaneously in *B. stephanieae*. We find that sperm exchange, and therefore male function in *B. stephanieae* begins between 8 and 10 wpf (6–8 weeks before laying egg masses) and between 6 and 9 mm in length (Fig. 6). The ovotestis begins to produce sperm before sperm exchange starts, as spermatogenesis is evident in small (6 mm) juveniles (Fig. 5G). Our results show that sexual development is more complex than existing descriptions of simultaneous hermaphroditism in nudibranchs would suggest and contributes detailed data about the timing and allocation of sexual resources.

### Sperm exchange occurs before egg laying

Here, we provide detailed evidence that individuals of *B. stephanieae* become functionally male before becoming simultaneous hermaphrodites. While making direct comparisons between the time- and size-based experiments is difficult (see below), animals exchange sperm when they are at least 6 weeks younger and 3 mm smaller than when they are able to lay eggs (Fig. 6). Sperm exchange and subsequent production of fertilized egg masses occurs in animals 9 mm and larger, suggesting that the size classes defined in our experiments correspond roughly to immature juveniles (small juveniles, 6 mm), which do not exchange or store sperm; males (large juveniles, 9 mm), which exchange and store sperm; and adult hermaphrodites (adults, >12 mm, which exchange and store sperm and immediately produce fertilized eggs. While spermatogenesis is evident in immature juveniles (Fig. 5G), their inability to exchange sperm indicates that they are not yet functional males. This is consistent with previous work in the nudibranch *Phestilla sibogae*, which showed the size of animals able to exchange sperm (5–8 mg) is 10–20x smaller than those able to lay eggs (90–480 mg, Todd et al. 1997). Other groups of heterobranch gastropods show a similar pattern of reproductive development. Juveniles of the sacoglossan *Alderia modesta* exchange sperm before being able to lay eggs (2 versus 10 days postmetamorphosis) at half the size (0.5 mm versus 1.2 mm long) (Angeloni 2003), and in the sacoglossan *Elysia maoria*, male gonads appear in animals only 5 mm long, while female gonads begin to appear in animals 10 mm long (Reid 1964). Mating experiments in the cephalaspid *Mariaglaja tsurugensis* (=*Chelidonura sandrana*) using body weight as a proxy for age also suggest that smaller, and therefore younger, animals behave primarily as males, although no egg laying activity was recorded to confirm reproductive function (Sprenger et al. 2009). Becoming functionally male before egg mass production begins appears to be more common in heterobranch gastropods than is widely recognized.

The phenomenon of a male phase before simultaneous hermaphroditism is best categorized as simultaneous hermaphroditism with adolescent protandry, a subset of simultaneous hermaphroditism (after Leonard 2018). Elsewhere, this pattern of male first reproduction has been described as adolescent gonochorism (Heller 1993), protandrous hermaphroditism (Wägele and Willan 2000), or “intermediate between the conventional “simultaneous” and “sequential” categories” (Todd et al. 1997). The term “protandrous hermaphroditism” implies a switch from one sex to the other including loss of function of the first sex, which is not the case here (Ghiselin 1969; Leonard 2018). This is also not likely an evolutionarily intermediate stage since sequential hermaphroditism does not appear to have evolved from simultaneous hermaphroditism in other gastropods (Collin 2013). Our results highlight the importance of documenting reproduction through the full reproductive life cycle. In order to draw conclusions about the mechanisms that underlie transitions between sexual systems, we must have a fuller understanding of reproductive allocation, particularly within simultaneous hermaphrodites (Schärer 2009; Nakadera and Koene 2013).

### Fertilization over time

We predicted that fertilization would decrease uniformly, starting high and then going to zero as an individual “ran out” of sperm. For example, in the nudibranch *Hermissenda crassicornis*, % fertilization decreased as more egg masses were produced within an individual (Rutowski 1983). Similarly, fertilization rates of singly mated individuals of the heterobranch *Aplysia californica* started at high levels and declined as egg mass production continued (Ludwig and Walsh 2004), and individuals of *Mariaglaja tsurugensis* showed declines in fertilization after 10 days in isolation (Anthes et al. 2006). While this was generally the case in our study, not all of our findings showed an immediate decline. Egg masses laid by the 2 animals isolated at 12 wpf show an initial increase in fertilization before decreasing, after producing approximately 10 egg masses (Fig. 3A and B), while large (9 mm) juveniles produced egg masses with high fertilization rates until around the 10th egg mass, at which point fertilization decreased significantly (Fig. 3C). While it is difficult to draw conclusions about these patterns based on the small sample size (*n* = 2, *n* = 4), fertilization rates for all individuals monitored eventually declined to zero. Unexpectedly, two individuals (8 wpf,  Fig 2C; J6xA,  Fig 4A) produced a single fertilized egg out of hundreds or thousands of eggs laid. No other animals in the same treatment groups laid fertilized eggs (Figs. 2 and 4). Self-fertilization is unlikely based on anatomy in this and other nudibranchs (Hadfield and Switzer-Dunlap 1984). More likely, low rates of sperm transfer or insufficient development of the seminal receptacle (Fig. 5D) to maintain sperm contributed to this rare occurrence. Sperm present in these animals may also have degraded over the 7–8 week period between isolation and laying. However, other individuals in our experiments are able to store sperm for months (below).

Precocious sperm exchange requires that individuals be able to store sperm for some period of time. Sperm storage duration varies widely across the animal kingdom, from a matter of hours to years (see Orr and Brennan 2015 for review). Across the 15 individuals tracked in our study, we observed storage times of several months (Fig. 3C). In the nudibranch *Aeolidiella glauca*, isolated individuals were able to lay fertilized egg masses for 5–6 weeks (Karlsson and Haase 2002), and controlled matings of laboratory-reared *Aplysia californica* showed that individuals were able to produce fertilized egg masses from a single mating bout for up to 41 days (Ludwig and Walsh 2004). Most studies do not track animals for long enough to estimate the maximum duration of sperm storage; therefore, the number of fertilized egg masses produced by an individual might be a useful proxy. For example, in the nudibranch *Hermissenda crassicornis*, a single mating event provided enough sperm to lay about 2–3 fertilized egg masses (Rutowski 1983), and a single mating event in *A. california* produced ∼9 fertilized egg masses (Ludwig and Walsh 2004). Rutowski used a cutoff of 50% fertilized eggs within a single egg mass to determine whether or not an egg mass is fertilized. Converting our data to match this cutoff, a week of mating in *B. stephanieae* was sufficient for an average of 14 fertilized egg masses. Obviously, more mating opportunities were available to individuals over the course of our experiment, though we did not quantify how many times animals mated. The ability to store sperm prior to the production of fertilized eggs and reciprocal mating opportunities produces high fecundity in individuals of *B. stephaniae*, similar to reports from other heterobranchs.

Generally speaking, long-term storage of sperm requires some physical adaptation (specific storage organ or otherwise) and may also include mechanisms for supporting and maintaining sperm (Orr and Brennan 2015). The reproductive system of nudibranchs is notoriously convoluted and complex, and in many species, the reproductive tract includes areas where sperm from other individuals (allosperm) is stored (Hadfield and Switzer-Dunlap 1984; Wägele and Willan 2000). Here, we see distinct areas of sperm with heads directed toward the wall of the reproductive tract, identifying the seminal receptacles (Fig. 5G–I). No obvious changes are seen in the epithelium of the seminal receptacles between 6 and 9 mm in juveniles (Fig. 5), but the appearance of sperm in both the seminal receptacles (allosperm) and ampulla (autosperm) in later-stage (9 mm and larger) animals indicates sperm is both given and received.

### Variation in fertilization across treatments

Proportion of fertilized embryos varied across treatments, with high rates of inter-individual variation in reproductive success. As previously noted, growth rates are highly variable in *B. stephaniae* and depend on environmental conditions (i.e., temperature, food availability, competition with conspecifics). This makes direct comparisons between age- and size-based experiments difficult, as well as suggesting a source of inter-individual variation. Overall, animals in the initial age-based experiments had much lower rates of fertilization compared to individuals in the subsequent size-based experiments, despite starting at about the same absolute age in both sets of experiments (compare Fig. 2C with Fig. 4A). These differences in fertilization are likely due to longer periods of sperm storage (2–4 weeks) in the initial age-based experiment, though may also have been due to preferable conditions (i.e., higher temperatures, greater food availability, no competition with conspecifics) in the follow-up size-based experiment. In all experiments, the proportion of fertilized embryos was markedly reduced in older animals. Most large (9 mm) juveniles consistently laid egg masses with very high levels of fertilization while most adult hermaphrodites laid egg masses with lower levels of fertilization (Fig. 4B). These adult animals were collected from our initial experiment, and at ∼7 months old, were ∼3 months older than the large juveniles (∼4 months old) at first laying. While age does not appear to affect sperm quality as animals lay egg masses of similar fertilization rates regardless of their mate (Fig. 4B), differences in fertilization between treatments may be due to senescence and a decrease in egg quality in older animals. Senescence after oviposition is common in nudibranchs, with many having lifespans of less than 6 months (Folino 1993; Schlesinger et al. 2009; Wolf and Young 2012). In our hands, the lifespan of *B. stephaniae* is between 6–18 months in the lab (data not shown). The lifespan of *B. stephaniae* is likely to be shorter in the wild. High predation on nudibranchs (Harris 1975) selects for early reproductive output, which is highly correlated with early senescence (Luckinbill et al. 1984). It should be noted that *B. stephanieae* has a long history of laboratory culture, which may also impact fertilization rates. More practically, this result suggests focusing mating schemes/experiments on young adults in laboratory culture to maximize the availability of high-quality reproductive material.

## Conclusions

Detailed studies of reproduction, including reproductive timing and histology, provide important information about life history and allow us to draw inferences about the lability of sexual systems. Similar studies in other species will be necessary to understand how widespread developmental variation in simultaneously hermaphroditic species is and to address how such systems may have evolved. Additionally, better understanding of the reproductive system of *B. stephanieae* will in turn enhance our ability to introduce genetic material in the *Berghia* system. For example, powerful new techniques such as ReMOT Control (Chaverra-Rodriguez et al. 2018, 2020) or DIPA-CRISPR (Shirai et al. 2022), which introduce transgenic components into adult animals, targeting the ovaries directly, will require a detailed understanding of reproductive processes in order to be effective. The increasing availability of genetic tools opens new avenues for exploring the diversity of sexual systems. However, such future work requires the solid foundations provided by detailed anatomical and reproductive data, such as what we provide here.

## Supplementary Material

obac034_Supplemental_File

## Data Availability

The data underlying this article are available on GitHub, at [https://github.com/lyons-lab/berghia_reproduction]. Histological slides and voucher specimens for the size-based experiments are available from the Scripps Institution of Oceanography Benthic Invertebrate Collection (SIO-BIC), under the catalog numbers M18647–M18659.
