# Supplementary material for: Precocious Sperm Exchange in the Simultaneously Hermaphroditic Nudibranch, Berghia stephanieae
Source: Integr Org Biol. 2022 Aug 1;4(1):obac030. doi: 10.1093/iob/obac034 (PMC9449679; doi:10.1093/iob/obac034)
Supplement: obac034_Supplemental_File [file obac034_supplemental_file.docx]

**Supplemental File 1**

*Feeding Schedule for Time-Based Isolation Experiment*

About 3 weeks after they were laid, a group of roughly 300 early juveniles were initially fed 1-5 small *E. diaphana* (<1 mm pedal disk diameter, <5 mm column height) every other day. For the first two weeks of feeding, two small *E. diaphana* were fed every other day to the 300 juveniles. From 2 to 4 wpf, groups of 11 were fed 1 small *E. diaphana* every other day. From 4 to 5 wpf, groups of 9 were fed 2 small *E. diaphana* every other day. After 5 wpf, groups were fed one-sixth of a medium *E. diaphana* (2-4 mm pedal disk diameter, 10-25 mm column height) for the remainder of the experiment. Isolated juveniles were fed 2 medium *E. diaphana* tentacles each every other day from 4 to 8 wpf. From 8 to 12 wpf, isolated juveniles were fed 3 medium *E. diaphana* tentacles every other day. After 12 wpf, isolated juveniles were fed one-twelfth of a medium *E. diaphana*.

**Supplemental Table 1.** Individuals of *Berghia stephanieae* in the time-based isolation experiment were fed every other day.

| Age | Group Feeding  (average # of individual *B. stephanieae*) | Isolated individuals |
| --- | --- | --- |
| 0-2 wpf | 1-5 small *E. diaphana* (~300) |  |
| 2-4 | 1 small *E. diaphana* (11) |  |
| 4-5 | 2 small *E. diaphana* (9) | 2 tentacles from medium *E. diaphana* |
| 5-6 | ⅙ medium *E. diaphana* (9) | 2 tentacles from medium *E. diaphana* |
| 6-8 | ⅙ medium *E. diaphana* (6) | 2 tentacles from medium *E. diaphana* |
| 8-10 | ⅙ medium *E. diaphana* (4) | 3 tentacles from medium *E. diaphana* |
| 10-12 | ⅙ medium *E. diaphana* (3) | 3 tentacles from medium *E. diaphana* |
| 12+ | ⅙ medium *E. diaphana* (2) | 1/12 medium *E. diaphana* |

**
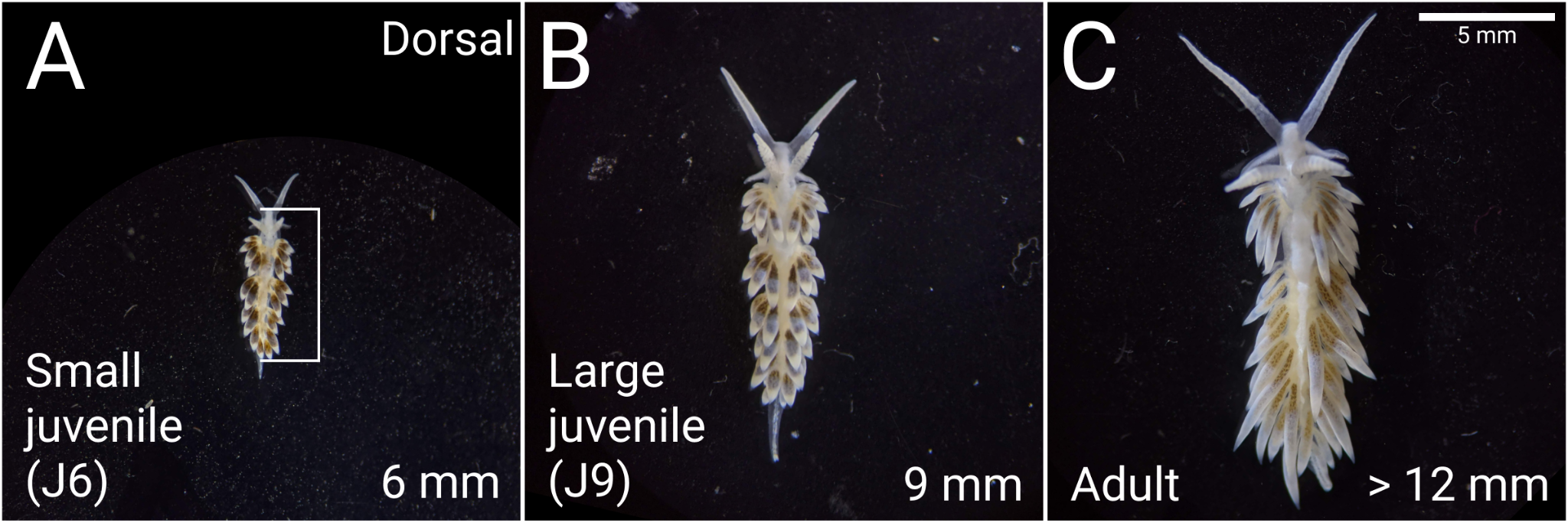
**

**Supplemental Figure 1. Size categories of *Berghia stephanieae*. A-C.** Dorsal view of *B. stephanieae* at 3 lengths measured from mouth to end of cerata. Measurements for all individuals were made from the anterior-most part of the head to the most posterior cerata as indicated in **A**. Small juveniles (6 mm) are unable to exchange sperm, and produce unfertilized egg masses after several more weeks of growth. **B.** Large juveniles (9 mm) function as males, and are able to exchange and store sperm, producing primarily fertilized egg masses after a few weeks of additional growth. **C.** Adults are mature hermaphrodites (~12-20 mm), able to exchange and store sperm, and produce fertilized egg masses immediately. Scale bar = 5 mm.
